# Conscious Self-regulation, Motivational Factors, and Personality Traits as Predictors of Students’ Academic Performance: A Linear Empirical Model

**DOI:** 10.11621/pir.2022.0411

**Published:** 2022-12-30

**Authors:** Varvara I. Morosanova, Irina N. Bondarenko, Tatiana G. Fomina

**Affiliations:** a Psychological Institute of the Russian Academy of Education, Moscow, Russia; b Russian Academy of Education, Moscow, Russia

**Keywords:** Conscious self-regulation (SR), engagement, motivation, personality, academic achievement

## Abstract

**Background:**

The theoretical basis of this study was the resource approach (Morosanova 2014, 2017), in which the conscious self-regulation of learning activity is understood as a meta-resource for students, allowing them to consciously and independently set learning goals and manage their achievement. This approach made it possible to create models of direct and mediate contributions of self-regulation and school engagement not only to academic performance, but also to other motivational and personal competencies.

**Objective:**

Our study aimed to investigate the impact of conscious self-regulation, school engagement, motivation, and personality on academic achievement, while taking into account the effects of mediation.

**Design:**

A quantitative research design was applied, using data collected from more than 1524 students from the 5^th^ to 11^th^ grades in Russian schools and applying Structural Equation Modelling (SEM).

**Results:**

The results allowed us to construct a statistical model of predictors of students’ academic achievement. The model was verified on the total sample, as well as samples differing in gender and age. The results show that conscious self-regulation is central to non-cognitive predictors of academic achievement. For the first time, a study has revealed and described the reciprocal relationship between self-regulation, academic motivation, school engagement, and academic performance. The resulting model demonstrates that behavioral and cognitive engagement make a significant contribution to academic performance, while emotional and social engagement do not find significant links with it, although they determine other areas of school life.

**Conclusion:**

Our paper investigates the nature and strength of the effects of major non-cognitive predictors of academic achievement. The study results substantiated the resource role of conscious self-regulation not only for students’ academic performance, but also for their academic motivation, school engagement, and attitude toward learning. The predictor model of academic achievement we developed will provide a foundation for combining existing heterogeneous concepts into a single integrated model and clarify the contradictions between them.

## Introduction

Conscious self-regulation, school engagement, and academic motivation are considered the most significant factors advancing academic performance (Morosanova, Fomina, & Bondarenko, 2015; Gordeeva, Sychev, Gizhitsky, & Gavrichenkova, 2017; Steinmayr, Weidinger, Schwinger, & Spinath, 2019). The literature suggests that determinants of academic achievement range from personality characteristics (traits, intelligence, engagement, motivation, experience, and attitudes) to school factors (peer and teacher support, autonomy, and available resources) (Petrides, Chamorro-Premuzic, Frederickson, & Furnham, 2005). While priority has previously been given to cognitive characteristics such as intelligence and executive functions (EF) (Deary, Strand, Smith, & Fernandes, 2007; Zelazo, Blair, & Willoughby, 2016), now researchers are increasingly turning to non-cognitive predictors (*e.g*., Richardson, Abraham, & Bond, 2012).

The evidence that non-cognitive predictors (in particular, self-regulation, motivation, engagement, and emotional attitude toward learning) directly contribute to academic performance has been reliably replicated across samples that differ in age, gender, type of school activity, cultural differences, and so on. However, the specifics of the relationship between the non-cognitive predictors and their joint impact on academic success have not been studied sufficiently, although researchers emphasize the relevance of integrative models of non-cognitive predictors of student academic achievement (for example, Mega, Ronconi, & De Beni, 2014). Constructing comprehensive models allows researchers to take into account the versatile relationship that arises between the variables. So, the present study had as its objective to build structural models of the relationships between conscious self-regulation, motivational factors, and personality traits, and academic success, as well as to assess the moderating and mediating effects of their joint contribution.

### Self-Regulated Learning as a Predictor of Academic Achievement

Self-regulated learning (SRL) has been one of the main topics in educational psychology research for several decades. In the general field of scientific research, methods for studying self-regulation have been developed in the frameworks of different approaches, and naturally complement each other. In its most general form, SRL is defined as a complex phenomenon ensuring the maintenance of the cognitive and motivational processes that contribute to the development of conscious behavior to achieve learning goals (Pandero, 2017). The present study is based on the original approach, which considered conscious self-regulation a reflexive means of setting goals (including educational ones) and managing their achievement. According to this approach, the multicomponent structure of self-regulation determines its general development, which, in turn, serves as a meta-resource for students; it predicts their academic success and also contributes to school engagement and psychological well-being (Morosanova, 2021).

The results of our research are in line with the global trend, according to which the study of self-regulation makes it possible to explain individual differences in students’ academic achievements, thereby proving the possibilities of self-regulation in advancing academic performance (*e.g.,* Dent & Koenka, 2016, etc.). High SRL is associated with better academic performance, with high-achieving students using its defining strategies more frequently and effectively than their lower-achieving peers (Zimmerman, 2002).

### School Engagement as a Predictor of Academic Achievement

During the last 10 years, researchers have shown great interest in the “school engagement” construct. Following the competent approaches, we consider school engagement to be the sustainable, directed, active participation of students in educational activities and in school life in general, manifested through their behavior, and cognitive and emotional involvement, as well as features of their social interaction in the academic environment (Wang, Degol, & Henry, 2019). It has been shown that students with a high level of school engagement are characterized by having more effective learning strategies, coping more successfully with learning difficulties, and being more likely to achieve their educational goals (Fredricks, Filsecker, & Lawson, 2016). Students who are more engaged in school have been shown to have higher academic performance (Chase et al., 2014).

School engagement is a multifaceted concept. Researchers have identified several components, such as behavioral, emotional, cognitive, social, etc. (Fredricks, Blumenfeld, & Paris, 2004; Wang, Shim, & Wolters, 2017). The effect of student engagement on academic performance varies depending on the components of engagement that are examined. According to some studies, highly significant relationships are found between academic achievement and a student’s general level of school engagement (Fomina et al., 2020), as well as its particular components: cognitive and behavioral engagement (Fredricks et al., 2004).

### Motivation as a Predictor of Academic Achievement

Different motivational theories and constructs have been put forward to try to understand how and why students are motivated for academic achievement (Pintrich, 2003). The problem of learning motivation’s impact on academic success is widely represented in studies (Olivier et al., 2019; Schnitzler, Holzberger, & Seidel, 2021). According to meta-analyses, intrinsic academic motivation is the strongest predictor of academic achievement: students with intrinsic motivation have significantly higher levels of academic achievement and engagement than those with predominantly extrinsic motivation (Richardson et al., 2012).

In this article, we focus on the motivational constructs that appear to be mainly associated with SRL and play an essential role in supporting academic performance: cognitive motivation, attitude towards learning, and achievement motivation. Our study used the Ryan and Deci approach (self-determination theory, or SDT), which identifies the mechanisms of the functioning of extrinsic and intrinsic motivation (Ryan & Deci, 2012). Intrinsic motivation is associated with engagement in the educational process and higher achievement. It occurs when a person does something simply because this activity gives him pleasure; external reinforcement is not needed. At the same time, extrinsic and intrinsic motivation do not exist separately from each other; there are mutual transitions between them.

### Personality as a Predictor of Academic Achievement

The personality traits included in the Big Five are most often considered to be intrapersonal predictors of academic success. To date, a lot of data has been accumulated in this domain, which has been systematized and summarized in the framework of meta-analyses (*e.g.,* O’Connor & Paunonen, 2007).

Among the Big Five,Conscientiousness has the closest relationship with academic performance (Dumfart & Neubauer, 2016). This is the only trait whose impact on academic performance is comparable to (and according to some data, even exceeds) that of cognitive abilities (Richardson et al., 2012). The research on Extraversion’s effect on academic performance shows ambiguous results. The relationship between Neuroticism and academic achievement is usually negative. Openness to Experience has a strong relationship with intelligence and is therefore positively associated with learning success. Most studies show no significant relationship between Agreeableness and academic performance (according to the meta-analysis of O’Connor & Paunonen, 2007). It has been demonstrated that there are regulatory bases for the personality dispositions, while conscious self-regulation has stable links with Conscientiousness, which, with a high development of SR, ensures high academic performance and compensates for the multidirectional influence of Extraversion and Neuroticism (Morosanova, 2021).

All the phenomena discussed above have been well studied in terms of their impact on academic achievement, but their mutual influence on each other has not been adequately considered. This creates the need for developing structural models and verifying them empirically.

Essentially, the current study aims to provide the answers to the following questions:

RQ 1: What is the nature of the relationship between conscious self-regulation, school engagement, motivation, and personality traits as predictors of students’ academic achievement?

RQ 2: What is the specificity of these predictors in determining students’ academic achievement?

In this regard, the following hypotheses were tested:

Hypothesis H1: There is a significant relationship between self-regulation, academic motivation, and school engagement. It is expected that this relationship is reciprocal in nature.

As shown above, there are some specifics in the joint influence of self-regulation, academic motivation, school engagement, and attitude toward learning on academic performance. We designed another group of hypotheses to uncover this specificity.

Hypothesis H2a: Personality traits are expected to show a positive linear relationship with school engagement and self-regulation, as well as a predictive effect for academic achievement;

Hypothesis H2b: School engagement is expected to mediate the relationship between self-regulation and academic achievement;

Hypothesis H2c: A student’s emotional-motivational attitude toward learning is expected to positively correlate with school engagement and positively contribute to academic achievement.

**Figure 1.**
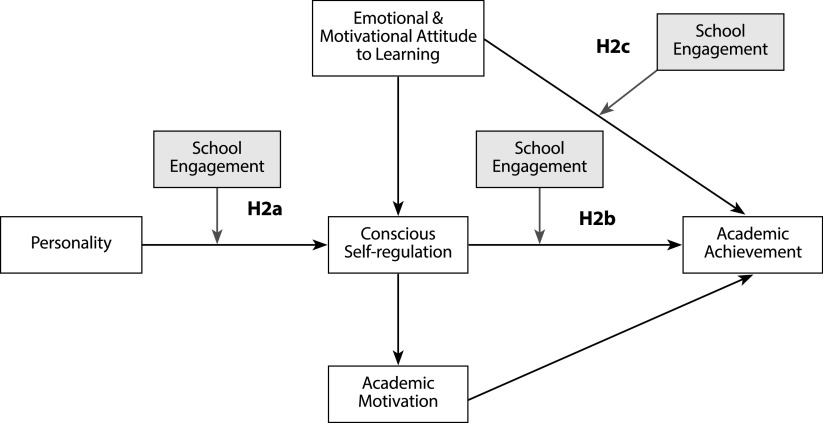
Theoretical framework (mediating effects of school engagement)

Schematically, these hypotheses are presented in *Figure 1*.

## Methods

### Participants

To test the proposed hypotheses and provide answers to the research questions, the current study adopted a quantitative research design; data was collected in surveys which were conducted with the help of self-administered questionnaires. Respondents were students in the 5^th^ to 11^th^ grades (N = 1524) from Moscow and the Moscow region.

### Procedure

#### Self-Regulated Learning

For assessment of the students’ conscious self-regulation development the “Self-Regulation Profile of Learning Activity Questionnaire — SRPLAQ” was used (Morosanova & Bondarenko, 2017). It includes 67 statements describing typical situations of achieving educational goals. These generate 10 scales corresponding to the structural components of the conscious self-regulation:

1. Planning (*e.g.,* “I often try to set a certain amount of time needed to complete the learning task”);
2. Modeling (*e.g*., “Unexpected changes in the timetable throw me off my stride”);
3. Programming (*e.g.,* “When preparing for a test (exam), I usually think over the order of studying the material”);
4. Results evaluation (*e.g*., “Even when I’m tired, I tend to study until I’m satisfied with the result”);
5. Flexibility (*e.g*., “If I need to get prepared for a lesson, I can work even in an uncomfortable and unfamiliar situation”);
6. Independence (*e.g.,* “I use every opportunity to make reports in class”);
7. Reliability (*e.g*., “I do not postpone preparing for the lessons even if I’m tired or feel sick”);
8. Responsibility (*e.g.,* “I do not give up preparing for the lessons even if I have to choose between studying and spending time with my peers”);
9. Social desirability (*e.g*., “I always admit my mistakes”); and
10. The general level of self-regulation (total score for all scales).

The SRPLAQ was previously validated in a sample of 14 to 18-year-old students (N = 702). The validation study demonstrated that the coefficients of internal consistency of items for each scale ranged from 0.58 to 0.76, indicating an overall reasonable homogeneity of the items in each scale. The subscales were significantly correlated with each other (r = 0.22–0.66, p < 0.001). Each statement is rated on a 4-point scale (Yes — Probably Yes – Probably No — No). The responses are then reduced to only “yes” and “no,” by counting “probably yes/probably no” as “yes/no” respectively. The “yes” responses are then added up (items are reversed if necessary), so that high scores (maximum 9 for each scale) denote high self-regulation.

#### Academic motivation

Participants completed the Scales of Academic Motivation in School-Age Children (Gordeeva et al., 2017). The Russian edition was developed on the basis of The Academic Motivation Scale (AMS-C), which has its theoretical foundation in self-determination theory and its sub-theory of intrinsic and extrinsic motivation. The questionnaire contains 32 statements, including three scales for assessing intrinsic learning motivation (cognitive motivation, achievement motivation, self-development motivation); four scales for assessing extrinsic motivation (external motivation (parents), external motivation (general), introjected motivation, self-respect motivation); and amotivation. The responses for all statements were measured with a 5-point Likert scale ranging from 1 (“Strongly Disagree”) to 5 (“Strongly Agree”).

The attitudes toward learning in middle and high school were identified by means of the Spielberger’s “State-Trait Personality Inventory, STPI” as modified by Bondarenko (Bondarenko et al., 2018). We used three Spielberger’s subscales: anxiety, anger, and curiosity. The depression scale was replaced by the achievement motivation scale. The Russian edition of the inventory includes 40 statements generating four scales and is designed to diagnose a student’s emotional and motivational attitude toward learning. Responses for the variables are measured with a 5-point Likert scale ranging from 1 (“Strongly Disagree”) to 5 (“Strongly Agree”). It results in an overall score called the general level of attitude toward learning (AL).

#### School Engagement

School engagement has been assessed by means of the Multidimensional School Engagement Scale (Wang et al., 2019, Russian adaptation by Fomina & Morosanova, 2020). The questionnaire evaluates behavioral, cognitive, emotional, and social components of school engagement/disengagement. It includes eight scales and contains 37 statements, which are evaluated by subjects on a 5-point Likert scale, with answers ranging from 1 (“Not like me at all”) to 5 (“Very similar to me”). The scales have acceptable reliability (Cronbach’s alpha from 0.63 to 0.90). Confirmatory factor analysis confirmed the preservation of the original bifactorial structure of the questionnaire with the identification of two global factors of engagement and disengagement. In the context of the research objectives, an integral scale of school engagement was used.

#### Personality

We assessed personality traits by means of the Russian version of the “Big Five Questionnaire — Children (BFQ-C)” (Malykh, Tikhomirova, & Vasin, 2015). The questionnaire contains 62 statements. Items rated on a 5-point scale, with response options ranging from 1 (“Strongly Disagree”) to 5 (“Strongly Agree”), and measures five personality factors: Extraversion, Openness, Agreeableness, Neuroticism (or Emotional Stability as a positive pole), and Conscientiousness.

#### Academic achievement

The average score of annual grades in Russian language and Mathematics was used as an indicator of academic performance.

SPSS 26.0 (SPSS Inc.) was used to obtain descriptive statistics for the study variables and bivariate associations. We performed SEM in AMOS 23 to verify our hypotheses.

## Results

The Pearson correlations, means, and σ coefficients of the variables under study are reported in *Table 1*.

**Table 1:** Descriptive Statistics and Inter-scale Correlations (N = 1524)

		M±σ	1	2	3	4	5	6	7	8	9	10	11	12	13
1	Academic Achievement	4.06±0.7	1												
2	Self-regulation	27.80±8.9	.20	1											
3	Emotional Attitude to Learning	0.00±13.5	.24	.64	1										
4	Motivation	3.61±0.9	.25	.53	.61	1									
5	Extraversion	45.10±8.3	.10	.32	.46	.35	1								
6	Agreeableness	46.54±8.5	.13	.37	.50	.44	.63	1							
7	Conscientious ness	43.38±8.3	.17	.59	.60	.54	.54	.67	1						
8	Neuroticism	31.44±9.6	–.12	–.47	–.58	–.30	–.14	–.21	–.32	1					
9	Openness	45.95±7.8	.29	.50	.58	.57	.56	.58	.67	–.2	1				
10	Engagement (Overall score)	68.05±13.4	.18	.55	.64	.63	.54	.61	.63	–.3	.59	1			
11	Behaviour Engagement	13.66±3.4	.21	.49	.54	.55	.41	.47	.55	–.2	.56	.81	1		
12	Cognitive Engagement	18.17±4.2	.21	.56	.56	.55	.37	.46	.62	–.3	.52	.77	.60	1	
13	Emotional Engagement	18.09±4.3	.10	.38	.52	.56	.43	.49	.48	–.3	.43	.85	.57	.52	1
14	Social Engagement	18.13±4.5	.10	.38	.47	.41	.55	.57	.44	–.2	.45	.83	.56	.46	.65

*Note. p<0.01*.

**Figure 2.**
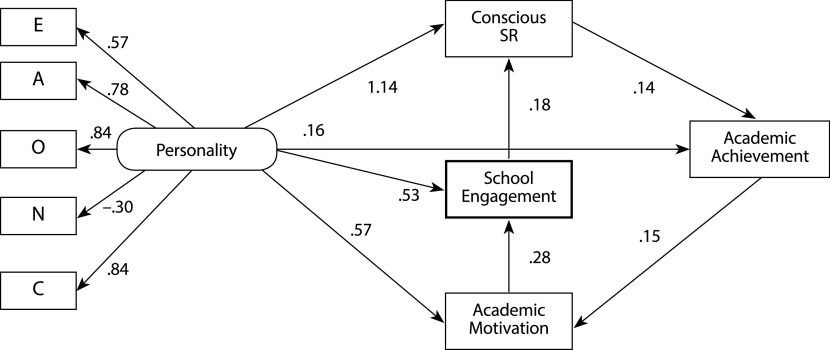
Path Diagram of the Proposed Model of the Present Study (Overall Scores). Model 1

*Note. E = Extraversion, O = Openness, A = Agreeableness, N = Neuroticism, C = Conscientiousness*.

Path analysis was undertaken through AMOS, and the structural model was constructed to show the overall measures of main factors (*Figure 2*).

Model 1, made for the integral indicators, is shown in *Figure 2*. It has good statistical agreement with the theoretical model (χ^2^/*df* = 2.57, *p* = .00, CFI= .99, GFI= .99, RMSEA = .037, PCLOSE = .94). It demonstrated that SR makes a direct significant contribution to academic achievement. In addition, the positive impact of SR on academic achievement was enhanced by engagement and a cademic motivation. The contribution of academic motivation to academic achievement was mediative in nature, through school engagement and self-regulation. Finally, the achieved results (grades) supported and enhanced the adolescents’ motivation to learn. In addition, all personality traits contributed positively to self-regulation, engagement, motivation, and academic achievement. Thus, Model 1 has confirmed the H1 hypothesis that there is a significant relationship between self-regulation, academic motivation, and school engagement. It also showed that this relationship is reciprocal in nature.

The second model (Model 2) shows particular components of school engagement. In addition, it is complemented with an indicator of the student’s emotional and motivational attitude toward learning in order to identify the role of emotional engagement in the determination of academic performance.

**Figure 3.**
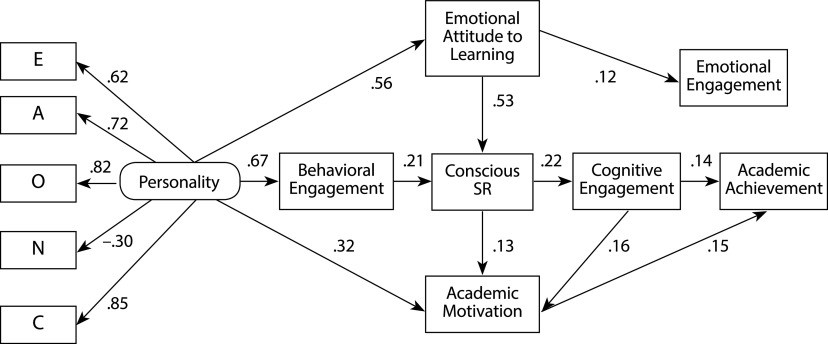
Path Diagram of the Proposed Model of the Present Study. Model 2.

*Note. E = Extraversion, O = Openness, A = Agreeableness, N = Neuroticism, C = Conscientiousness*.

The path diagram for the whole sample, along with standardized path coefficients of direct effects, is presented in *Figure 3*. It shows a very good fit to the data (χ^2^/*df* = 1.90, *p* = .00, CFI= .99, GFI= .99, RMSEA = .029, PCLOSE = .99). Our expectation that personality traits would show a positive linear relationship with school engagement and self-regulation, as well as a predictive effect on academic achievement, was confirmed completely (Hypothesis H2a).

Model 2 demonstrates that SR’s contribution to academic achievement is mediated by the cognitive engagement, which fully confirms the hypothesis of its mediator role in the relationship between self-regulation and academic achievement (Hypothesis H2b). Thus, conscious self-regulation, combining the influence of personality traits, the emotional and motivational attitude toward learning, and behavioral engagement, acts as a “stabilizing” resource for academic achievement, compensating for negative intrapersonal and emotional influences. It supports cognitive motivation and is enhanced by cognitive engagement.

The assumption that a student’s emotional-motivational attitude toward learning positively correlates with school engagement and makes a positive contribution to academic achievement was only partially confirmed. Indeed, an emotional-motivational attitude toward learning makes a positive contribution to emotional engagement, but emotional engagement itself does not make a significant positive contribution to academic achievement.

Thus, the model has revealed that the construct of school engagement is not holistic. However, its positive impact is included in the contribution of each of its significant success factors. Detailed information on the path coefficients and significance levels for the considered achievement factors is given in *Table 2*.

**Table 2:** Path coefficients and significance level

Path	R^2^	p value	Conclusion
Self-regulation → Academic motivation → School engagement → Academic achievement	0.27	0.000	H1 is supported
Personality → Behavior engagement → Self-regulation → Cognitive engagement → Academic achievement	0.32	0.000	H2a is supported
Self-(Cognitive regulation engagement → Academic as mediator) achievement	0.27	0.000	H2b is supported
Emotional Attitude to education → Academic Achievement (Emotional engagement as mediator)	0.24	0.000	H2c is partially supported

### Modifications of the general model for boys and girls and for different classes

Verification of the model on samples of students of different ages and gender demonstrated its invariant character (See *Table 3*); its components and the relationships between them were preserved for the subjects from the 5th to the 10th grade. In the 9th and 11th grades, the scores of the fit indices slightly decreased due to the reduced contribution of cognitive engagement to academic performance. This is due to the students’ preparation for passing the Unified State Examination (at the ends of grades 9 and 11), when children have to repeat the material already studied.

**Table 3:** Correspondence indices of models made for groups of students differing in gender and age

Model	N	χ^2^ / df	P	GFI	CFI	RMSEA	PCLOSE
Model 1 (grade 5)	187	1.43	.057	.96	.99	.048	.540
Model 2 (grade 6)	103	1.06	.693	.95	.99	.000	.916
Model 3 (grade 7)	105	.92	.584	.96	1,00	.000	.856
Model 4 (grade 8)	265	1.13	.273	.97	.99	.023	.928
Model 5 (grade 9)	151	1.83	.008	.95	.99	.074	.122
Model 6 (grade 10)	237	1.28	.42	.98	.99	.034	.776
Model 7 (grade 11)	39	1.78	.83	.91	.92	.143	.014
Model _F (girls)	471	1.05	.76	.99	1.00	.011	.992
Model _M (boys)	615	1.4	.12	.99	.99	.026	.965

Structural modeling allowed us to assess the contribution of conscious self-regulation to cognitive engagement (average β = .20). At the same time, cognitive engagement resulted in adding an average of 14% to the determination of academic achievement. In the 9th and 11th grades, due to the reason cited above, its contribution was reduced to 5%. Thus, the study results demonstrated the great importance of maintaining school engagement as a mediator that makes a significant contribution to academic performance in “quiet” periods of schooling.

The structural models built separately for boys and girls, on the whole, did not detect gender specificity in the determination of academic performance by the regulatory, motivational, and personal predictors. However, in female samples, researchers have traditionally recorded higher values for the contribution of self-regulation to engagement and engagement to academic performance. Girls are more organized, manage their time better, and use metacognitive strategies (Ruffing et al., 2015), consistent with the fact that girls tend to perform better during adolescence. Also, for girls, the predictive value of intrinsic motivation is higher at all levels of education, while for boys, the effects of external regulation are stronger (Vecchione et al., 2014). Boys are more likely than girls to demonstrate their indifference and ostentatious disregard for school norms and rules, while girls are more likely to transfer this to the emotional sphere (Kessels et al., 2014).

## Discussion

The novelty of the integrative model of non-cognitive predictors of academic performance constructed and verified in this study is not only determined by the composition of the considered predictors. Our study also provided new data on more complex patterns in the relationship between non-cognitive and academic performance compared to previous studies. Based on the data obtained, conscious self-regulation can be considered a core component of academic success, making not only a direct contribution to academic performance, but also supporting factors that contribute to the achievement of educational goals.

For the first time, a study has established the reciprocal nature of the relationship between self-regulation (SR), academic motivation, school engagement, and academic achievement (Hypothesis H1). Previously, some researchers showed a reciprocal relationship between SR and engagement (Karabenick & Zusho, 2015), while other researchers noted that these concepts are closely interrelated and complementary to each other (Cleary & Zimmerman, 2012). The present study has unambiguously confirmed its existence. This result allows us to speak about the resource role of conscious SR in relation to other significant predictors of academic success.

The reciprocal nature of the relationship between school engagement and self-regulation was also confirmed by the longitudinal research data: depending on the age of students, either self-regulation predicted higher levels of engagement, or vice versa (*e.g*., Fomina et al., 2021; Stefansson et al., 2018). Thus, in adolescence, self-regulation compensated for the lack of engagement and motivation of students, maintaining optimal levels of academic achievement (Bakracevic Vukman, & Licardo, 2010). On the other hand, the higher the students’ engagement, the higher their need to achieve educational goals. Thus, for the first time, it has been clearly shown that engagement in this case is a means of maintaining and developing self-regulation which eventually lays the foundation for successful learning. At the same time, it was shown that self-regulation was positively associated with productive forms of intrinsic motivation in all age groups: the more motivated a student was, the more effective his self-regulation (Rheinberg, Vollmeyer, & Rollett, 2000).

Hypothesis H2a, which asserted that personality traits will show a positive linear relationship with school engagement and self-regulation, thus influencing academic performance, has also been confirmed. School engagement in this case was represented by behavioral and cognitive components. On the whole, little is known about the relationship between personality characteristics and school engagement (Moreira et al., 2021). Most of the research in this direction has been carried out with the samples of older age (students and adults). Thus, it has now been shown that agreeableness and conscientiousness are reliable predictors of cognitive engagement; extraversion, agreeableness, conscientiousness, and openness predict behavioral engagement; and agreeableness is predictive of emotional engagement (Qureshi et al., 2016).

As for the relationship between self-regulation and personality characteristics, this issue has been well studied. Students with a high level of self-regulation development have certain personality structures: low neuroticism combined with high openness to experience, high extraversion, agreeableness, and conscientiousness (*e.g.,* Dörrenbächer & Perels, 2016). According to the data from previous studies, conscious self-regulation compensates for personality traits that hinder high performance (Morosanova, 2021). Studies have also shown that increasing the level of conscious self-regulation development helps to compensate for extreme expressions of personality characteristics. Thus, for example, introverts with a higher level of SR development have higher levels of openness to new experience. In this way, conscious self-regulation is actually a direct determining factor in academic performance, while personality features determine some individual peculiarities of students’ academic behavior. This issue needs further research.

Hypothesis H2b, which concerned the mediating role of cognitive school engagement in the relationship between self-regulation and academic achievement, has been confirmed. The conceptual contiguity of self-regulation and cognitive engagement is emphasized by a number of researchers (*e.g*., Ben-Eliyahu et al., 2018). Our data clearly indicate that conscious self-regulation is a resource for not only academic achievement, but also for school engagement. Indeed, progressing toward one’s internal goal (consciously relevant to one’s true needs and motivations) encourages students to be more engaged in the educational activities (Vasalampi et al., 2009). In general education schools, significant relationships are found between all types of school engagement and self-regulation (Wang, Deng, & Du, 2018). Longitudinal data show a reciprocal relationship between self-regulation and engagement in high school as well (Stefansson et al., 2018).

Hypothesis H2c, which posited that the emotional-motivational attitude toward learning is positively correlated with school engagement and makes a positive contribution to academic performance, found only partial confirmation in this study. Indeed, some factors of the emotional-motivational attitude toward learning, such as cognitive activity, achievement motivation, and negative emotions of anger and anxiety, make but little contribution to emotional engagement. However, contrary to the hypothesis, emotional engagement itself is not significantly associated with academic performance.

At present, the issue of including emotional factors in multifactorial models of academic success still remains under discussion. Researchers pay great attention to the regulation of emotions, especially maintaining their positive and negative balance at an optimal level (McRae & Gross, 2020). Thus, the ambiguous contribution of positive emotions to academic performance is known (for example, Pekrun et al., 2017). It is noted that the high intensity of positive emotions can act as one of the factors hindering the achievement of learning goals (Sallquist et al., 2009) due to a decrease in volitional control and the emergence of behavioral problems. It is possible to conclude that emotional engagement will probably make a significant contribution to another important indicator of schooling - the subjective well-being of students. But this hypothesis requires further testing.

## Conclusion

The research results allowed us to construct a new integrative model of predictors of students’ academic achievement. The data generated by this model show that conscious self-regulation is central to non-cognitive predictors of academic performance. The resource role of SR is thus substantiated, not only in determining academic performance, but also in relation to motivation, engagement, and attitude toward learning. For the first time, a reciprocal relationship between conscious self-regulation, academic motivation, school engagement, and academic performance has been described. It has been shown that behavioral and cognitive engagement make a significant contribution to academic performance, while emotional and social engagement did not have significant links with it, although they determine other areas of school life.

The theoretical contribution of the present study to the research topic lies in its possible clarification of the nature of the components of school engagement. The research results demonstrated that the school engagement construct is not holistic. Thus, its behavioral component is more of a personality formation, while the cognitive one is closer to regulatory-cognitive properties. The model of non-cognitive predictors of academic performance obtained in the study has demonstrated its invariant nature; it could be reproduced on samples that differed in gender and age. This model has revealed the role and place of conscious self-regulation as a key resource for academic success, regulating the activation of its other predictors.

The data obtained in this study provides essential results for pedagogical practice, psychological counseling, and psychoprophylactic work at school in the direction of advancing students’ academic performance. The resource role of conscious self-regulation is emphasized as both a direct predictor of academic performance and a factor regulating the positive impact of such achievement predictors as motivation, school engagement, and the emotional attitude toward learning.

## Limitations

The limitation of this study is the model’s lack of an intelligence indicator. Analysis of academic performance, as a rule, provides for an assessment of the impact of intelligence. The results of numerous studies have shown that its impact varies widely and on average explains up to 25% of the variance of the annual score. We did not include intelligence in this study because we aimed to investigate the impact of so-called non-cognitive predictors of academic achievement (conscious self-regulation, school engagement, motivation, and personality), taking into account the effects of mediation. In followup, we plan to include intelligence indicators in the model.

## References

[ref1] Bakracevic Vukman, K., & Licardo, M. (2010). How cognitive, metacognitive, motivational and emotional self-regulation influence school performance in adolescence and early adulthood. Educational Studies, 36(3), 259–268. 10.1080/03055690903180376

[ref2] Ben-Eliyahu, A., Moore, D., Dorph, R., & Schunn, C.D. (2018). Investigating the multidimensionality of engagement: Affective, behavioral, and cognitive engagement across science activities and contexts. Contemporary Educational Psychology, 53, 87–105. 10.1016/j.cedpsych.2018.01.002

[ref3] Bondarenko, I.N., Tsyganov, I.Yu., & Morosanova, V.I. (2018). Faktornaia struktura oprosnika “Otnoshenie k ucheniiu v srednikh i starshikh klassakh shkoly” [Factor structure of the “Attitude towards learning in middle and high school “ questionnaire]. In E.I. Gorbacheva, E.Yu. Savin, K.V. Kabanov, & O.N. Bakurova (Eds.), Lichnost’, intellekt, metakognitsii: issledovatel’skie podkhody i obrazovatel’nye praktiki. Materialy III-y Mezhdunarodnoi nauchno-prakticheskoi konferentsii 19–21 aprelya 2018 g. Kaluga, Rossiya (pp. 3–9). IP Yakunin A.V.

[ref4] Chase, P.A., Hilliard, L.J., John Geldhof, G., Warren, D.J.A., & Lerner, R.M. (2014). Academic achievement in the high school years: The changing role of school engagement. Journal of Youth and Adolescence, 43(6), 884–896. 10.1007/s10964-013-0085-4 24477498

[ref5] Cleary, T.J., & Zimmerman, B.J. (2012). A cyclical self-regulatory account of student engagement: Theoretical foundations and applications. In S.L. Christenson, A.L. Reschly, C. Wylie (Eds.) Handbook of Research on Student Engagement (pp. 237–257). Springer New York. 10.1007/978-1-4614-2018-7_11

[ref6] Deary, I., Strand, S., Smith, P., & Fernandes, C. (2007). Intelligence and educational achievement. Intelligence, 35(1), 13–21. 10.1016/j.intell.2006.02.001

[ref7] Deci, E.L., & Ryan, R.M. (2012). Motivation, personality, and development within embedded social contexts: An overview of self-determination theory. In R.M. Ryan (Ed.), The Oxford Handbook of Human Motivation (pp. 85–107). Oxford University Press.

[ref8] Dent, A.L., & Koenka, A.C. (2016). The relation between self-regulated learning and academic achievement across childhood and adolescence: A meta-analysis. Educational Psychology Review, 28(3), 425–474. 10.1007/s10648-015-9320-8

[ref9] Dörrenbächer, L., & Perels, F. (2016). Self-regulated learning profiles in college students: Their relationship to achievement, personality, and the effectiveness of an intervention to foster self-regulated learning. Learning and Individual Differences, 51, 229–241. 10.1016/j.lindif.2016.09.015

[ref10] Dumfart, B., & Neubauer, A.C. (2016). Conscientiousness is the most powerful noncognitive predictor of school achievement in adolescents. Journal of Individual Differences, 37(1), 8–15. 10.1027/1614-0001/a000182

[ref11] Fomina, T.G., & Morosanova, V.I. (2020). Adaptatsiia i validizatsiia shkal oprosnika “Mnogomernaia shkala shkol’noi vovlechennosti” [Russian adaptation and validation of the “Multidimensional School Engagement Scale”]. Vestnik Moskovskogo universiteta. Seriia 14. Psikhologiia [Moscow University Psychology Bulletin], 3, 194–213. 10.11621/vsp.2020.03.09

[ref12] Fomina, T.G., Filippova, E.V., & Morosanova, V.I. (2021). Longitiudnoe issledovanie vzaimosviazi osoznannoy samoreguliatsii, shkol’noi vovlechennosti i akademicheskoi uspevayemosti uchashchikhsia [Longitudinal study of the relationship between conscious self-regulation, school engagement and student academic achievement]. Psikhologicheskaia nauka i obrazovanie [Psychological Science and Education], 26(5), 30–42. 10.17759/pse.2021260503

[ref13] Fredricks, J.A., Blumenfeld, P.C., & Paris, A.H. (2004). School engagement: Potential of the concept, state of the evidence. Review of Educational Research, 74(1), 59–109. https://doi.org/10.3102%2F00346543074001059

[ref14] Fredricks, J.A., Filsecker, M., & Lawson, M.A. (2016). Student engagement, context, and adjustment: Addressing definitional, measurement, and methodological issues. Learning and Instruction, 43, 1–4. 10.1016/j.learninstruc.2016.02.002

[ref15] Gordeeva, T.O., Sychev, O.A., Gizhitsky, V.V., & Gavrichenkova, T.K. (2017). Shkaly vnutrennei i vneshnei akademicheskoi motivatsii shkol’nikov [Intrinsic and Extrinsic Academic Motivation Scale for Schoolchildren]. Psikhologicheskaia nauka i obrazovanie [Psychological Science and Education], 22(2), 65–74. 10.17759/pse.2017220206

[ref16] Karabenick, S.A., & Zusho, A. (2015). Examining approaches to research on self-regulated learning: Conceptual and methodological considerations. Metacognition and Learning, 10(1), 151–163. 10.1007/s11409-015-9137-3

[ref17] Kessels, U., Heyder, A., Latsch, M., & Hannover, B. (2014). How gender differences in academic engagement relate to students’ gender identity. Educational Research, 56(2), 220–229. doi.org/10.1080/00131881.2014.898916">http://dx.doi.org/10.1080/00131881.2014.898916

[ref18] Lei, H., Cui, Y., & Zhou, W. (2018). Relationships between student engagement and academic achievement: A meta-analysis. Social Behavior and Personality: An International Journal, 46(3), 517–528. 10.2224/sbp.7054

[ref19] Malykh, S.B., Tikhomirova, T.N., & Vasin, G.M. (2015). Adaptatsiia russkoiazychnoi versii oprosnika “Bol’shaia Piaterka — detskii variant” [Adaptation of the Russian version of the “Big Five Questionnaire — Children (BFQ-C)”]. Teoreticheskaia i eksperimentalnaia psikhologiia [Theoretical and Experimental Psychology], 8(4), 6–12.

[ref20] McRae, K., & Gross, J.J. (2020). Emotion regulation. Emotion, 20(1), 1–9. 10.1037/emo0000703 31961170

[ref21] Mega, C., Ronconi, L., & De Beni, R. (2014). What makes a good student? How emotions, self-regulated learning, and motivation contribute to academic achievement. Journal of Educational Psychology, 106(1), 121–131. 10.1037/a0033546

[ref22] Moreira, P.A., Inman, R.A., Cloninger, K., & Cloninger, C.R. (2021). Student engagement with school and personality: a biopsychosocial and person-centered approach. British Journal of Educational Psychology, 91(2), 691–713. 10.1111/bjep.12388 33247604

[ref23] Morosanova, V.I., Fomina, T.G., & Bondarenko, I.N. (2015). Academic achievement: Intelligence, regulatory, and cognitive predictors. Psychology in Russia: State of the Art, 8(3), 136–157. 10.11621/pir.2015.0311

[ref24] Morosanova, V.I., & Bondarenko, I.N. (2017). Diagnostika osoznannoi samoreguliatsii uchebnoi deiatel’nosti: novaia versiia oprosnika SSUD-M [Diagnosis of conscious self-regulation of educational activity: a new version of the SRPLAQ questionnaire]. Teoreticheskaia i eksperimental’naia psikhologiia [Thheoretical and experimental psychology], 10(2), 27–37.

[ref25] O’Connor, M.C., & Paunonen, S.V. (2007). Big Five personality predictors of post-secondary academic performance. Personality and Individual Differences, 43(5), 971–990. 10.1016/j.paid.2007.03.017

[ref26] Olivier, E., Archambault, I., De Clercq, M., & Galand, B. (2019). Student self-efficacy, classroom engagement, and academic achievement: Comparing three theoretical frameworks. Journal of Youth and Adolescence, 48(2), 326–340. 10.1007/s10964-018-0952-0 30421327

[ref27] Panadero, E. (2017). A review of self-regulated learning: Six models and four directions for research. Frontiers in Psychology, 8, Article 422. 10.3389/fpsyg.2017.00422 PMC5408091 28503157

[ref28] Pintrich, P.R. (2003). A Motivational Science Perspective on the Role of Student Motivation in Learning and Teaching Contexts. Journal of Educational Psychology, 95(4), 667–686. 10.1037/0022-0663.95.4.667

[ref29] Pekrun, R., Lichtenfeld, S., Marsh, H.W., Murayama, K., & Goetz, T. (2017). Achievement emotions and academic performance: Longitudinal models of reciprocal effects. Child Development, 88(5), 1653–1670. 10.1111/cdev.12704 28176309

[ref30] Petrides, K.V., Chamorro-Premuzic, T., Frederickson, N., & Furnham, A. (2005). Explaining individual differences in scholastic behaviour and achievement. The British Journal of Educational Psychology, 75(2), 239–255. 10.1348/000709904X24735 16033665

[ref31] Qureshi, A., Wall, H., Humphries, J., & Bahrami Balani, A. (2016). Can personality traits modulate student engagement with learning and their attitude to employability? Learning and Individual Differences, 51, 349–358. 10.1016/j.lindif.2016.08.026

[ref32] Rheinberg, F., Vollmeyer, R., & Rollett, W. (2000). Motivation and action in self-regulated learning. In M. Boekaerts, P.R. Pintrich, & M. Zeidner, Handbook on self-regulation. Directions and challenges for future research. Academic Press.

[ref33] Richardson, M., Abraham, C., & Bond, R. (2012). Psychological correlates of university students’ academic performance: a systematic review and meta-analysis. Psychological Bulletin, 138(2), 353–387. 10.1037/a0026838 22352812

[ref34] Ruffing, S., Wach, F., Spinath, F.M., Brünken, R., & Karbach, J. (2015). Learning strategies and general cognitive ability as predictors of gender-specific academic achievement. Frontiers in Psychology, 6, 1238. 10.3389/fpsyg.2015.01238 26347698 PMC4541601

[ref35] Sallquist, J.V., Eisenberg, N., Spinrad, T.L., Reiser, M., Hofer, C., Zhou, Q., ...Eggum, N. (2009). Positive and negative emotionality: Trajectories across six years and relations with social competence. Emotion, 9, 15–28. 10.1037/a0013970 19186913 PMC2753671

[ref36] Schnitzler, K., Holzberger, D., & Seidel, T. (2021). All better than being disengaged: Student engagement patterns and their relations to academic self-concept and achievement. European Journal of Psychology of Education, 36(3), 627–652. 10.1007/s10212-020-00500-6

[ref37] Stefansson, K.K., Gestsdottir, S., Birgisdottir, F., & Lerner, R.M. (2018). School engagement and intentional self-regulation: A reciprocal relation in adolescence. Journal of Adolescence, 64, 23–33. 10.1016/j.adolescence.2018.01.005 29408096

[ref38] Steinmayr, R., Weidinger, A.F., Schwinger, M., & Spinath, B. (2019). The importance of students’ motivation for their academic achievement — Replicating and extending previous findings. Frontiers in Psychology, 10, Article 1730. 10.3389/fpsyg.2019.01730 PMC6685139 31417459

[ref39] Vasalampi, K., Salmela-Aro, K., & Nurmi, J.E. (2009). Adolescents’ self-concordance, school engagement, and burnout predict their educational trajectories. European Psychologist, 14(4), 332–341. 10.1027/1016-9040.14.4.332

[ref40] Vecchione, M., Alessandri, G., & Marsicano, G. (2014). Academic motivation predicts educational attainment: Does gender make a difference? Learning and Individual Differences, 32, 124–131. 10.1016/j.lindif.2014.01.003

[ref41] Wang, C., Shim, S.S., & Wolters, C.A. (2017). Achievement goals, motivational self-talk, and academic engagement among Chinese students. Asia Pacific Education Review, 18(3), 295–307. 47(3), 633–662. 10.1007/s12564-017-9495-4

[ref42] Wang, M., Deng, X., & Du, X. (2018). Harsh parenting and academic achievement in Chinese adolescents: Potential mediating roles of effortful control and classroom engagement. Journal of School Psychology, 67, 16–30. 10.1016/j.jsp.2017.09.002 29571531

[ref43] Wang, M.-T., Degol, J.L., & Henry, D.A. (2019). An integrative development-in-sociocultural-context model for children’s engagement in learning. American Psychologist, 74(9), 1086–1102. 10.1037/amp0000522 31829690

[ref44] Wang, M.-T., Fredricks, J., Ye, F., Hofk ens, T., & Linn, J.S. (2019). Conceptualization and assessment of adolescents’ engagement and disengagement in school: A Multidimensional School Engagement Scale. European Journal of Psychological Assessment, 35(4), 592–606. 10.1027/1015-5759/a000431

[ref45] Zelazo, P.D., Blair, C.B., & Willoughby, M.T. (2016). Executive Function: Implications for Education. (NCER 2017–2000). National Center for Education Research, Institute of Education Sciences, U.S. Department of Education.

[ref46] Zimmerman, B.J. (2002). Becoming a self-regulated learner: an overview. Theory into Practice, 41, 64–70. 10.1207/s15430421tip4102_2

